# Cost-effectiveness analysis of penpulimab combined with paclitaxel and carboplatin as first-line treatment for advanced squamous non-small cell lung cancer

**DOI:** 10.3389/fphar.2025.1563788

**Published:** 2025-03-19

**Authors:** Xiaoyan You, Jiali Qin, Xiaomei Wang, Xianying Wang

**Affiliations:** ^1^ College of Pharmacy, Hebei Medical University, Shijiazhuang, China; ^2^ Department of Pharmacy, Hebei Medical University Third Hospital, Shijiazhuang, China

**Keywords:** non-small cell lung cancer, penpulimab, partitioned survival model, cost-effectiveness analysis, pharmacoeconomic evaluation

## Abstract

**Background:**

Advanced or metastatic squamous non-small cell lung cancer (NSCLC) represents a significant clinical and economic burden globally. In China, the introduction of innovative immunotherapy agents, such as penpulimab, has the potential to improve patient outcomes, but their high cost raises questions about affordability and cost-effectiveness.

**Objective:**

This study evaluates the economic viability of penpulimab combined with paclitaxel and carboplatin as a first-line treatment for this patient population.

**Methods:**

Data were obtained from the published randomized controlled trial AK105-302. A three-state partitioned survival model was developed to estimate the cost-effectiveness of the two treatments. One-way deterministic sensitivity analysis, probabilistic sensitivity analysis, and scenario analyses were performed to assess the robustness of the results and explore variations in key parameters.

**Results:**

The incremental cost-effectiveness ratio (ICER) for the penpulimab group compared to the placebo group was $16,105.90 per quality-adjusted life year (QALY), which falls below the willingness-to-pay (WTP) threshold of $37,709.46 per QALY. Deterministic sensitivity analysis identified the three most influential factors affecting model outcomes: discount rate, costs associated with progressive disease, and utility value for progression-free survival. Probabilistic sensitivity analysis indicated that at a WTP threshold of $37,709.46 per QALY, the probability of penpulimab being cost-effective reached 99%. Scenario analyses demonstrated that, while the base-case results were generally robust, the cost-effectiveness of penpulimab remained sensitive to the limited maturity of overall survival (OS) data in the penpulimab group. The immaturity of the OS data increased the extrapolation uncertainty, which could potentially alter the economic conclusions.

**Conclusion:**

Penpulimab, in combination with paclitaxel and carboplatin, demonstrates a cost-effectiveness advantage over placebo as a first-line treatment for advanced or metastatic squamous NSCLC in China, provided that long-term survival benefits align with extrapolations from the base case model. These findings support its prioritization in clinical practice within the current WTP thresholds. However, the economic conclusions remain contingent on resolving the uncertainties associated with immature OS data and validating extrapolation assumptions through extended follow-up studies.

## 1 Introduction

Primary bronchial lung cancer, commonly referred to as lung cancer, often develops insidiously, with nonspecific early symptoms. As a result, most patients are diagnosed at advanced stages, missing the optimal window for curative treatment and facing poor prognoses (Blandin et al., 2017). According to the latest national cancer burden report from the Chinese National Cancer Center, lung cancer accounted for 1.06 million new cases and 733,300 deaths in 2022, making it the leading cause of cancer incidence and mortality in China (Han et al., 2024). Based on histology, lung cancer is classified into small cell lung cancer (SCLC) and non-small cell lung cancer (NSCLC), with NSCLC, including adenocarcinoma, squamous cell carcinoma, and large cell carcinoma, comprising approximately 85%–90% of all lung cancer cases (Woodard et al., 2016).

Platinum-based doublet chemotherapy has long been the standard treatment for advanced NSCLC; however, its overall survival (OS) rates remain unsatisfactory (Herbst et al., 2018). In recent years, the therapeutic paradigm for advanced NSCLC has evolved, with programmed cell death protein-1 (PD-1) and programmed death-ligand 1 (PD-L1) inhibitors emerging as first-line treatments for driver gene-negative advanced NSCLC. Current clinical guidelines recommend therapies such as pembrolizumab, camrelizumab, sugemalimab, toripalimab, sintilimab, and serplulimab (National Comprehensive Cancer Network, 2024). While these agents have demonstrated clinical efficacy, their widespread adoption remains limited by their high costs and economic burden. Numerous pharmacoeconomic studies have assessed the cost-effectiveness of these therapies, with findings suggesting that for Chinese patients with squamous NSCLC, domestically developed agents, such as sintilimab and toripalimab in combination with chemotherapy, may provide a more cost-effective alternative (Cheng et al., 2024). Recently, penpulimab, a novel PD-1 inhibitor independently developed in China, received approval from the China National Medical Products Administration in January 2023 for use in combination with paclitaxel and carboplatin as a first-line treatment for locally advanced or metastatic squamous NSCLC. This therapy has also been incorporated into the 2023 Chinese Society of Clinical Oncology (CSCO) Diagnosis and Treatment Guidelines for NSCLC (Chinese Society of Clinical Oncology, 2023).

The recommendation of penpulimab in combination with paclitaxel and carboplatin is supported by evidence from the AK105-302 (NCT03866993) clinical trial, a multicenter, randomized, double-blind, placebo-controlled phase 3 study conducted exclusively in a Chinese population (Zhong et al., 2024). The trial demonstrated a median progression-free survival (PFS) of 7.6 months in the penpulimab group, an improvement of 3.4 months over the placebo group, with a 57% reduction in the risk of disease progression or death (hazard ratio [HR]: 0.44). Additionally, the 30-month OS rate was 51.6% for the penpulimab group, compared to a median OS of 20.2 months in the placebo group (HR: 0.55).

Despite its clinical benefits, penpulimab has not yet been included in the Chinese National Reimbursement Drug List, and no economic evaluations have been conducted to assess its cost-effectiveness. Currently, sintilimab, toripalimab, and camrelizumab are included in the national reimbursement catalog. Therefore, this study aimed to develop a partitioned survival model from the perspective of the Chinese healthcare system to evaluate the cost-effectiveness of penpulimab in combination with paclitaxel and carboplatin as first-line treatment for locally advanced or metastatic squamous NSCLC in China. Additionally, it examined the potential economic value of penpulimab under the current reimbursement policy. The findings can generate evidence supporting the rational use of penpulimab in clinical practice and assess its value in improving outcomes in patients with NSCLC.

## 2 Materials and methods

### 2.1 Ethics approval

This study used publicly available data from the AK105-302 clinical trial and official sources. Ethical approval was not required as no human participants were directly involved, and no new data was collected.

### 2.2 Target population

The target population included individuals meeting the inclusion criteria outlined in the AK105-302 clinical trial. Eligible patients were aged 18–75 years with histologically or cytologically confirmed locally advanced (stage IIIb or IIIc) or metastatic (stage IV) squamous NSCLC, who were ineligible for surgical resection or concurrent/sequential chemoradiotherapy. Key inclusion criteria included an Eastern Cooperative Oncology Group (ECOG) performance status score of 0–1, no prior systemic chemotherapy for advanced NSCLC, at least one measurable lesion as defined by Response Evaluation Criteria in Solid Tumors, a life expectancy of ≥3 months, and normal organ function as indicated by screening laboratory results.

Exclusion criteria included patients with epidermal growth factor receptor (EGFR)-sensitive mutations or anaplastic lymphoma kinase (ALK) translocations, those who had previously received corresponding targeted therapies, and patients who had undergone systemic antitumor treatment or immunotherapy within 3 weeks before the trial’s start. Patients with mixed histology, including small cell lung cancer components, were excluded.

### 2.3 Intervention

According to the 2024 guidelines of CSCO for first-line treatment of stage IV squamous NSCLC, and based on the study design of AK105-302, we selected penpulimab in combination with paclitaxel and carboplatin as the experimental group, while the control group consisted of a placebo combined with paclitaxel and carboplatin. Patients were randomized into two groups to receive intravenous injections of 200 mg of penpulimab or placebo, combined with paclitaxel (175 mg/m^2^) and carboplatin (AUC 5), administered once every 3 weeks for four cycles. Following this initial phase, maintenance treatment with 200 mg of penpulimab or placebo was administered every 3 weeks. The carboplatin dose was calculated using the Calvert formula, capped at a maximum dose of 750 mg: Carboplatin dose (mg) = target AUC (mg/mL/min) × (creatinine clearance [mL/min] + 25).

Treatment continued until disease progression, unacceptable toxicity, lack of clinical benefit, withdrawal of consent, or completion of 24 months. In the penpulimab group, penpulimab monotherapy was continued beyond disease progression if deemed clinically beneficial by the investigator. Patients with confirmed disease progression were allowed to cross over to receive penpulimab in the placebo group. According to the AK105-302 trial data, 153 patients (87%) in the placebo group experienced disease progression or death, of whom 102 patients (58%) crossed over to penpulimab treatment. However, the trial did not report subsequent treatments for patients who did not receive penpulimab after progression.

To align with clinical guidelines, subsequent treatments were modeled based on the recommendations in the CSCO guidelines (Chinese Society of Clinical Oncology, 2024) and the Chinese Medical Association guideline (Oncology Society of Chinese Medical Association, 2024). These guidelines recommend second-line treatment with docetaxel monotherapy for patients progressing after first-line or maintenance therapy. To simplify the model, it was assumed that among placebo group patients who experienced disease progression, 102 would receive penpulimab monotherapy. In contrast, the remaining patients would switch to second-line docetaxel chemotherapy.

For the penpulimab group, a conservative assumption was made that all patients with disease progression would continue receiving penpulimab until exiting the trial or completing 24 months of treatment. Afterward, they would transition to second-line docetaxel therapy.

### 2.4 Model construction and survival analysis

This study used Microsoft Excel 2021 to develop a partitioned survival model for cost-effectiveness analysis. The model structure comprises three mutually exclusive health states: PFS, progressive disease (PD), and death. Patients enter the model in the PFS state and, over successive cycles, transition to the PD state or death (Woods et al., 2020).

The model operates with a 3-week cycle length, consistent with the treatment schedule in the AK105-302 trial. A 10-year time horizon was adopted, reflecting the low long-term survival rate for metastatic squamous NSCLC. According to the National Cancer Institute, the 10-year survival rate for advanced NSCLC is approximately 3.0% (Surveillance Research ProgramNational Cancer Institute, 2024). Based on extrapolated parametric survival models, the 10-year survival rates for the penpulimab and placebo groups are projected to be 14.0% and 4.7%, respectively.

Both costs and outcomes were discounted at an annual rate of 5%, as recommended by the guidelines for pharmacoeconomic evaluation (Chinese Pharmaceutical Association, 2024). Sensitivity analyses were conducted with discount rates ranging from 0% to 8% to test the robustness of the results.

In the partitioned survival model, survival data were used to estimate the number of patients in each health state per cycle. Kaplan-Meier survival curves for PFS and OS were used during the trial’s follow-up period. Survival data were extrapolated for time points beyond the follow-up period using parametric models. Data points from the PFS and OS curves in the AK105-302 trial were digitized using GetData Graph Digitizer 2.26, and individual patient data were reconstructed using the IPDfromKM package in R 4.4.1.

We applied standard parametric models, including exponential, Weibull, Gompertz, log-logistic, lognormal, generalized gamma, and gamma distributions, to fit the survival data. Additionally, the Royston-Parmar (RP) model was utilized as a flexible parametric approach for survival curve fitting and extrapolation. For the RP models, we assessed the best-fitting models across three scales: odds, normal, and hazard.

The model fit was evaluated using Akaike’s Information Criterion (AIC) and Bayesian Information Criterion (BIC), with lower values indicating better model performance (Latimer, 2013). A distribution was considered appropriate if its AIC value was within four points of the lowest AIC, whereas a BIC difference exceeding 10 from the lowest value was deemed unsuitable. Furthermore, when both statistical and visual assessments indicated that the same distribution adequately fit survival data for both groups, a common distribution was preferred over separate distributions for each group (Rothwell et al., 2021). The final selection of best-fitting distributions was based on a combination of statistical criteria and visual inspection (Table 1).

**TABLE 1:** AIC and BIC values for survival curve fitting distributions.

Criterion	Survival curve	Exponential	Gamma	Gompertz	Weibull	Log-logistic	Lognormal	Gengamma	RP-hazard	RP-odds	RP-normal
AIC	Penpulimab PFS curve	1632.900	1631.234	1632.537	1633.670	1613.988	1612.345	1611.889	1601.795	1601.552	**1601.308**
	Placebo PFS curve	1911.873	1876.722	1913.827	1894.735	1826.545	1849.245	1850.768	1739.512	1742.060	**1737.210**
	Penpulimab OS curve	1113.808	1112.667	1114.035	1112.754	**1112.644**	1115.255	1114.655	1114.728	1114.419	1116.306
	Placebo OS curve	1538.730	1530.365	1534.509	1530.660	**1530.558**	1537.288	1532.365	1532.450	1531.903	1531.771
BIC	Penpulimab PFS curve	1636.065	1637.564	1638.866	1640.000	1620.318	1618.674	1621.384	1617.619	1614.211	**1613.967**
	Placebo PFS curve	1915.037	1883.052	1920.157	1901.065	1832.874	1855.574	1860.263	1758.501	1761.049	**1756.199**
	Penpulimab OS curve	1116.972	1118.997	1120.365	1119.084	**1118.973**	1121.585	1124.149	1124.222	1123.913	1128.965
	Placebo OS curve	1541.895	1536.694	1540.839	1536.990	**1536.888**	1543.618	1541.859	1541.944	1541.398	1541.265

PFS, progression-free survival; PD, progression disease; AIC: Akaike’s Information Criterion; BIC: bayesian information criterion.

^*^Bold values indicate the AIC and BIC values of the distribution models selected for the base-case analysis.

**FIGURE 1:**
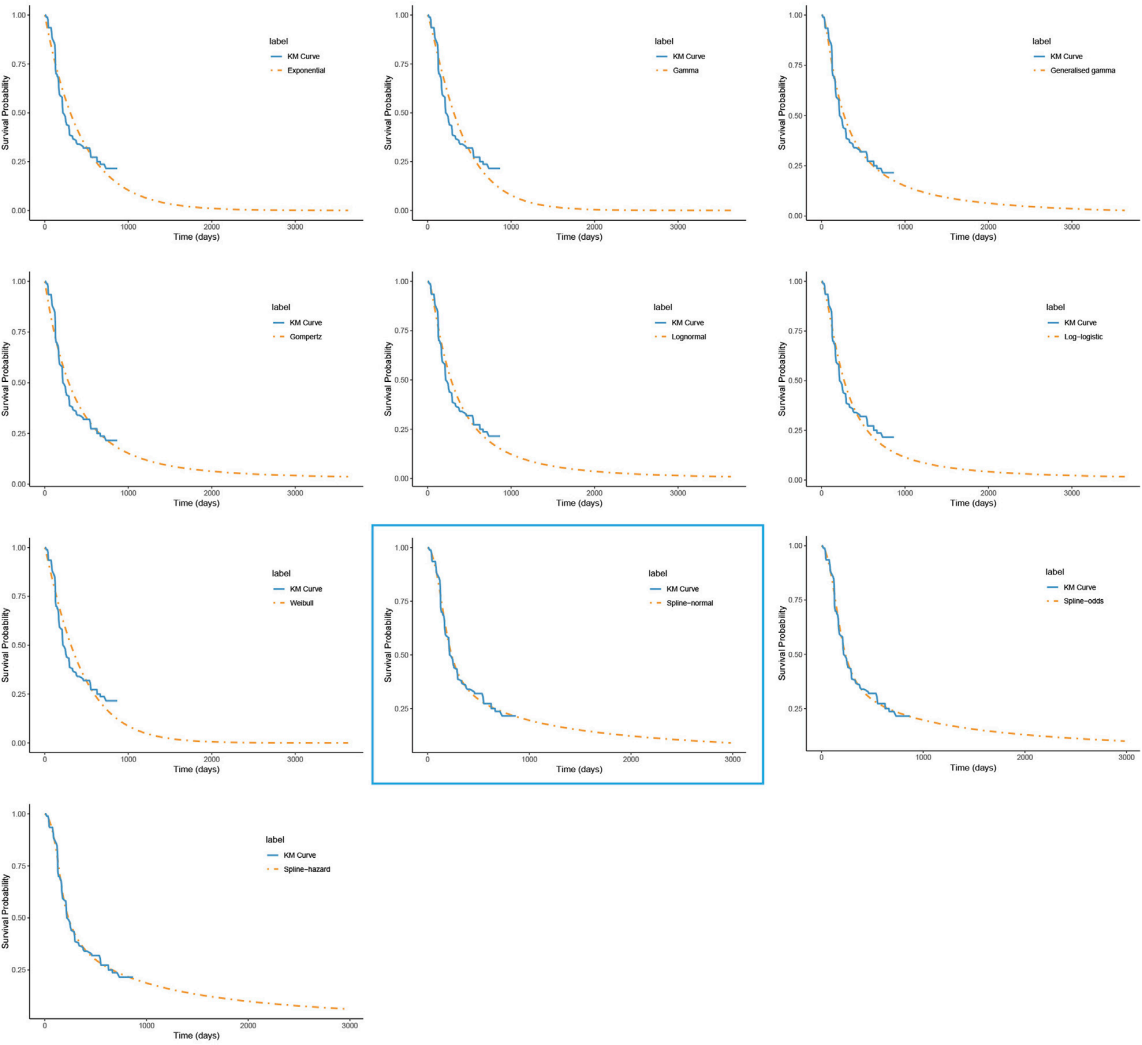
The fitted progression-free survival curves for the penpulimab group. *The distribution model selected in the blue box represents the one used in the base-case analysis..

**FIGURE 2:**
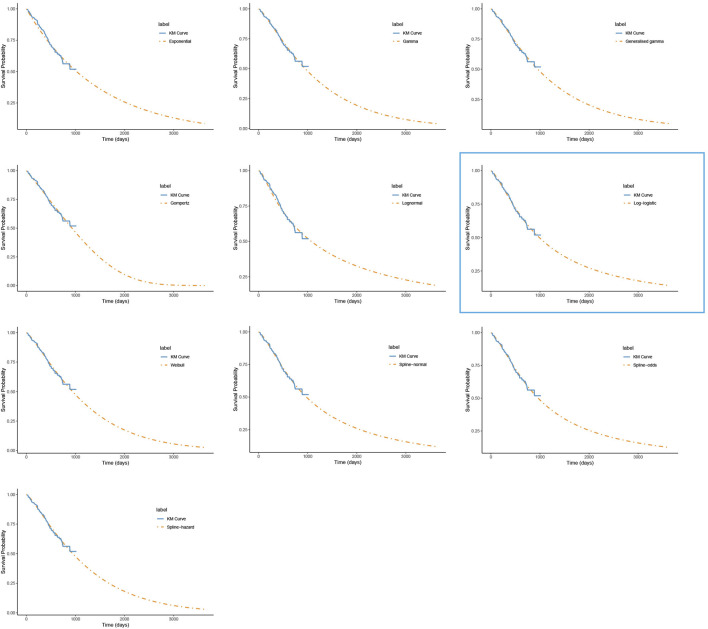
The fitted overall survival curves for the penpulimab group. *The distribution model selected in the blue box represents the one used in the base-case analysis.

**FIGURE 3:**
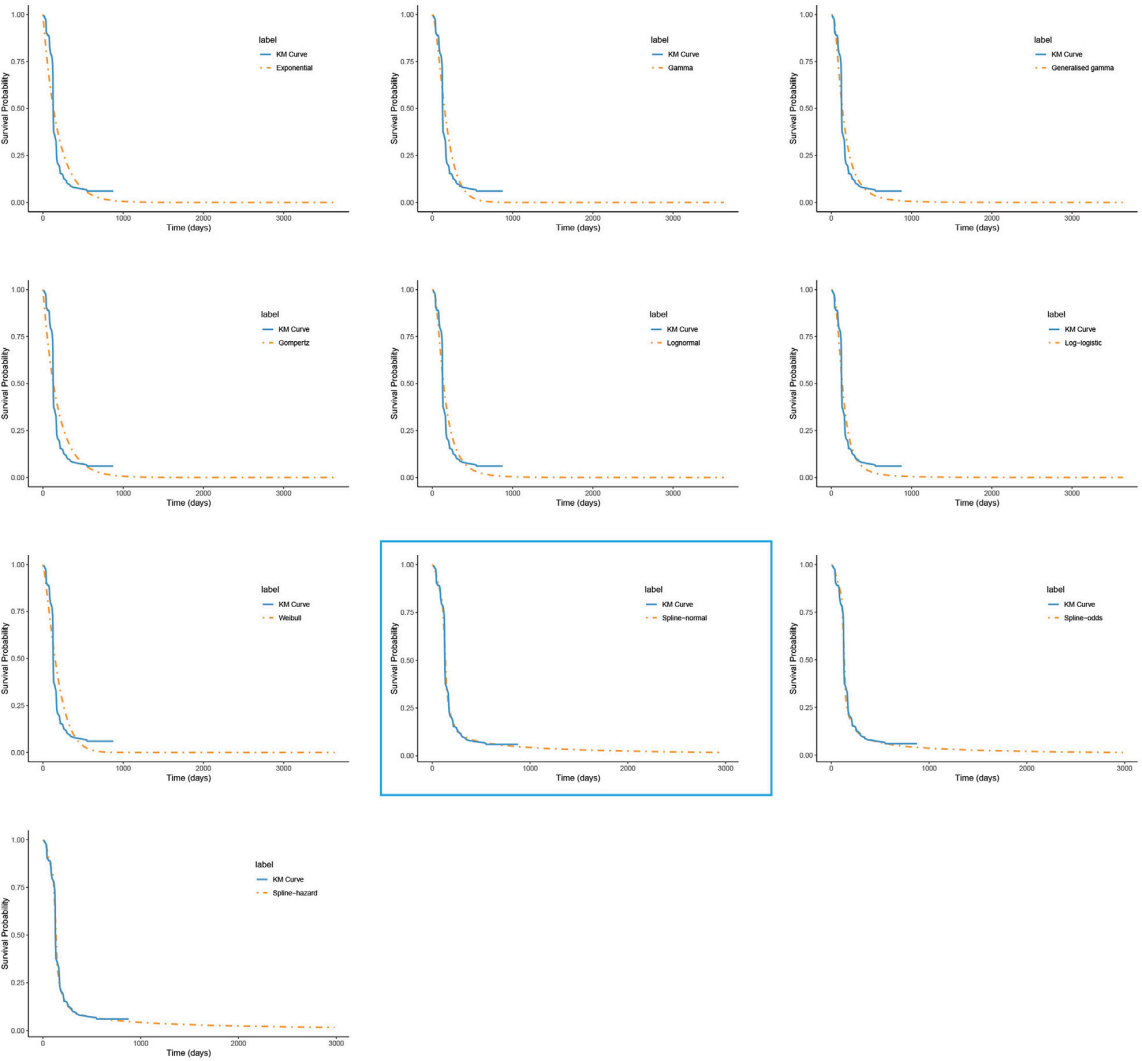
The fitted progression-free survival curves for the placebo group. *The distribution model selected in the blue box represents the one used in the base-case analysis.

**FIGURE 4:**
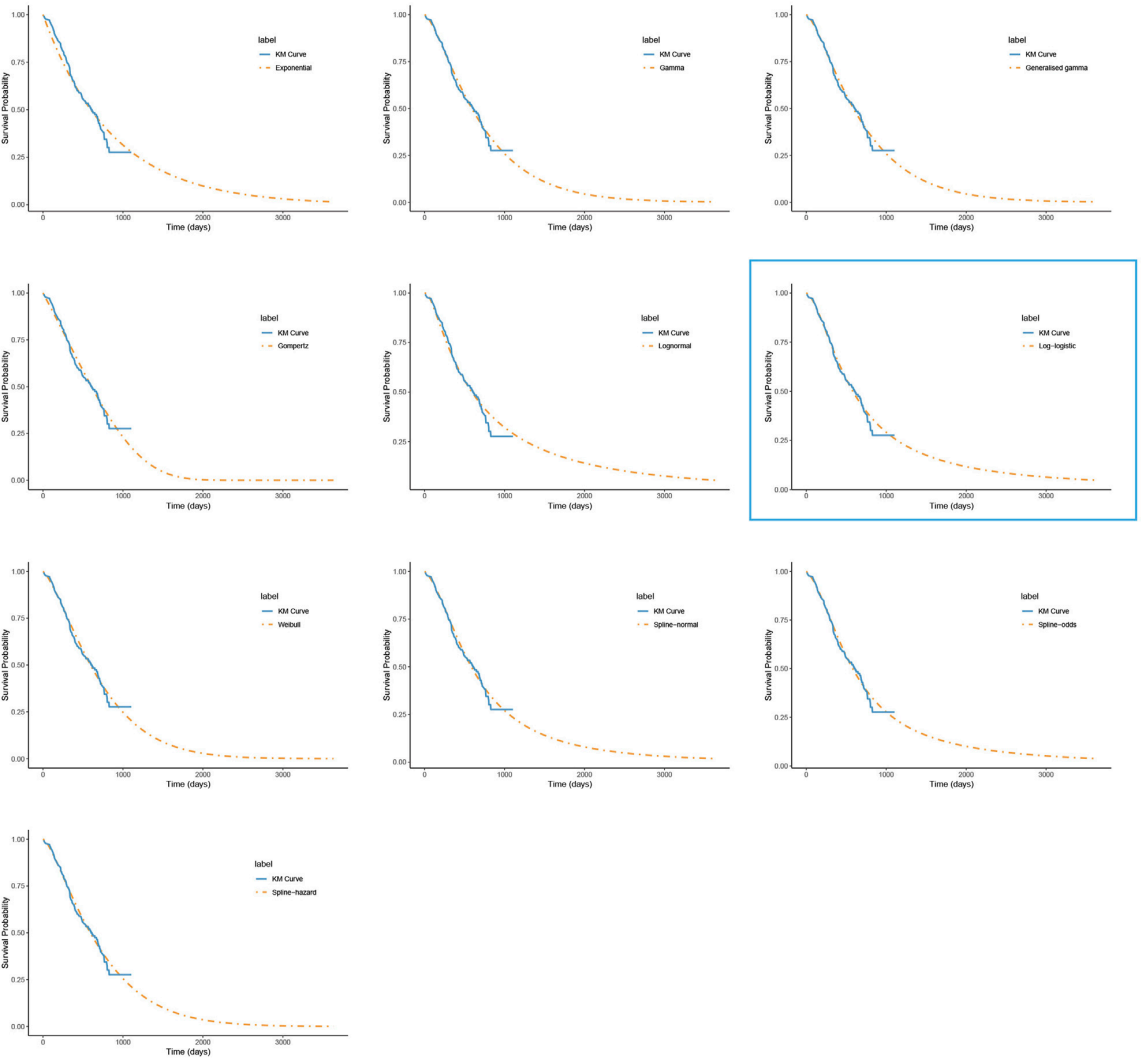
The fitted overall survival curves for the placebo group. *The distribution model selected in the blue box represents the one used in the base-case analysis.

For PFS, the RP-normal model was selected because of its superior fit to the data, while the log-logistic distribution was chosen for OS because it provided an optimal fit for both the penpulimab and placebo groups. Specifically, the log-logistic distribution was the second-best fitting model for the penpulimab group and the best-fitting model for the placebo group. The fitted PFS and OS curves for both the groups are presented in Figures 1–4, respectively. The distribution parameters used for the base-case analysis are listed in Table 2.

**TABLE 2:** Survival curve distributions and distribution parameters.

Survival curve	Distribution	Parameter value	
Penpulimab PFS curve	RP-normal	gamma0 = −4.29; gamma1 = 0.63; gamma2 = −0.56; gamma3 = 0.67; knot = 2; scale = normal	
Placebo PFS curve	RP-normal	gamma0 = −5.45; gamma1 = 1.05; gamma2 = 12.61; gamma3 = −301.26; gamma4 = 304.08; gamma5 = −15.16; knot = 2; scale = normal	
Penpulimab OS curve	Loglogistic	shape = 0.327	log scale = 985.622
Placebo OS curve	Loglogistic	shape = 0.496	log scale = 583.448

PFS: progression-free survival; PD: progression disease.

### 2.5 Costs and utility inputs

This study adopts the perspective of the Chinese healthcare system, focusing exclusively on direct medical costs. These include drug acquisition costs, disease management expenses, follow-up testing fees, end-of-life care costs, and adverse event management costs. The costs of penpulimab, paclitaxel, carboplatin, and docetaxel were derived from the median bid prices 2024 as reported in the Yaozh database (https://db.yaozh.com). Additional costs were sourced from published literature (Shao et al., 2022; Rui et al., 2022) and adjusted to 2024 values using the healthcare-related consumer price index (CPI).

Drug costs for the two treatment regimens were calculated based on dosages and treatment durations from the AK105-302 trial, assuming a simulated Chinese patient with a body surface area of 1.72 m^2^ and a creatinine clearance rate of 70 mL/min (Xiang et al., 2024). All costs were converted to a unified currency using the average exchange rate of 1 USD = 7.11 CNY, based on exchange rates from 1 January 2024, to 31 October 2024 (State Administration of Foreign Exchange, 2024).

Since the AK105-302 trial did not provide quality-of-life-related data, utility parameters were sourced from published literature (Rui et al., 2022; Shen et al., 2018). A utility value of 0.856 was assigned to the PFS state, and 0.768 was assigned to the PD state. These utility values were applied to estimate quality-adjusted life years (QALYs) over the 10-year model horizon. A detailed list of all input parameters, including costs and utility values, is provided in Table 3.

**TABLE 3:** Cost and utility parameters in the model.

Parameter	Dosage (mg/cycle)	Cost ($)/utility	Upper	Lower	Distribution	References
Cost Parameters						
Penpulimab	200 mg/3 weeks	502.19/100 mg	—	401.75	Gamma	Yaozh.com
Paclitaxel	301 mg/3 weeks	7.55/30 mg	68.83	1.95	Gamma	Yaozh.com
Carboplatin	475 mg/3 weeks	21.99/150 mg	41.36	10.89	Gamma	Yaozh.com
Docetaxel	129 mg/3 weeks	19.59/20 mg	128.01	3.18	Gamma	Yaozh.com
Cost of diagnosis	Every 3 weeks	3.24	4.81	1.60	Gamma	Shao et al. (2022)
Cost of intravenous injection	Every 3 weeks	1.78	2.21	1.60	Gamma	Shao et al. (2022)
Cost of nursing/day	3 days/3 weeks	3.89	4.61	3.07	Gamma	Shao et al. (2022)
Cost of bed/day	3 days/3 weeks	6.81	8.08	5.38	Gamma	Shao et al. (2022)
Cost of CT	Every 6 weeks for 54 weeks, then every 12 weeks	59.99	71.14	47.43	Gamma	Shao et al. (2022)
Cost of routine urine test	Every 3 weeks	0.65	0.77	0.52	Gamma	Shao et al. (2022)
Cost of blood routine examination	Every 3 weeks	3.24	3.85	2.57	Gamma	Shao et al. (2022)
Cost of blood biochemical test	Every 3 weeks	48.52	57.55	38.36	Gamma	Shao et al. (2022)
Cost of terminal care		2398.56	2878.27	1918.85	Gamma	Shao et al. (2022)
Adverse Event Parameter						
Anaemia		143.67	165.77	110.51	Gamma	Rui et al. (2022)
Neutrophil count decreased		119.08	370.48	52.92	Gamma	Rui et al. (2022)
White blood cell decreased		119.08	370.48	52.92	Gamma	Rui et al. (2022)
Platelet count decreased		1559.27	1834.44	1284.11	Gamma	Rui et al. (2022)
Utility Parameter						
PFS		0.856	0.994	0.718	Beta	Shen et al. (2018)
PD		0.768	0.941	0.595	Beta	Shen et al. (2018)
Health Utility Loss						
Anaemia		0.07	0.09	0.06	Beta	Rui et al. (2022)
Neutrophil count decreased		0.20	0.16	0.14	Beta	Rui et al. (2022)
White blood cell decreased		0.20	0.16	0.14	Beta	Rui et al. (2022)
Platelet count decreased		0.11	0.13	0.09	Beta	Rui et al. (2022)
Incidence of Adverse Events						
Penoulimab group						
Anaemia		0.017	0.021	0.014	Beta	Zhong et al. (2024)
Neutrophil count decreased		0.445	0.534	0.356	Beta	Zhong et al. (2024)
White blood cell decreased		0.202	0.243	0.162	Beta	Zhong et al. (2024)
Platelet count decreased		0.035	0.042	0.028	Beta	Zhong et al. (2024)
Placebo group						
Anaemia		0.080	0.096	0.064	Beta	Zhong et al. (2024)
Neutrophil count decreased		0.509	0.610	0.407	Beta	Zhong et al. (2024)
White blood cell decreased		0.206	0.247	0.165	Beta	Zhong et al. (2024)
Platelet count decreased		0.051	0.062	0.041	Beta	Zhong et al. (2024)
Survival Curve Parameters						
Body surface area (m^2^)		1.72	2.58	0.86	Normal	Xiang et al. (2024)
Creatinine clearance rate (mL/min)		70	105	35	Normal	Xiang et al. (2024)
Discount rate (%)		5	0	8	Fixed	Chinese Pharmaceutical Association (2024)

PFS, progression-free survival; PD, progression disease.

### 2.6 Adverse events

This study focuses exclusively on grade 3 or higher adverse events, as grade 1–2 events typically do not require clinical intervention. Adverse events with an incidence of 5% or more in any treatment group of the AK105-302 trial were included in the analysis. These events include anaemia, neutrophil count decreased, white blood cell decreased, and platelet count decreased (Zhong et al., 2024).

Grade 3 or higher adverse events often necessitate therapy discontinuation, dose adjustment, or additional medical interventions. For simplicity, the costs associated with managing these adverse events were modeled as one-time, derived from published literature, and adjusted for inflation using the healthcare-related CPI.

### 2.7 Uncertainty analysis

Based on our base-case assumptions, we conducted both one-way and probabilistic sensitivity analyses to assess the impact of parameter variations on the base-case results. Scenario analyses were performed to evaluate the effects of different assumptions on the model outcomes. The specific parameter variations considered in these analyses are listed in Table 3.

In the one-way sensitivity analysis, the upper and lower bounds of drug costs were determined based on the highest and lowest bid prices across different regions of China in 2024. For penpulimab, as it is a nationally unified exclusive drug with a low likelihood of future price increases, only a 20% price reduction was considered. Upper and lower bounds were derived from maximum and minimum values or 95% confidence intervals reported in the literature for other parameters. In cases where these were unavailable, a ±20% variation from the base value was applied. The results of the one-way sensitivity analysis are visualized using a tornado diagram.

A Monte Carlo simulation with 1,000 iterations was performed in the probabilistic sensitivity analysis. Key model parameters were simultaneously sampled from predefined distributions: gamma distributions for cost parameters, beta distributions for utility parameters and incidence rates, and normal distributions for body surface area and creatinine clearance (Xiang et al., 2024; Su et al., 2021). Results are displayed using cost-effectiveness scatter plots and cost-effectiveness acceptability curves.

Owing to the relatively short follow-up duration of the AK105-302 trial, the OS data remain immature. In our goodness-of-fit analysis, multiple models demonstrated a good fit, necessitating scenario analysis based on different OS curve distributions. Scenarios 1 and 2: For the penpulimab group, we selected the lognormal distribution (representing the best potential survival benefit) and the Gompertz distribution (representing the worst potential survival benefit). As the median OS for the penpulimab group has not yet been reached, the extrapolated survival rates varied significantly across different distribution models. Scenario 3: To further assess the survival benefits, we employed the Weibull distribution, which showed potential survival benefits and ranked just above the Gompertz distribution in terms of extrapolated survival duration. This scenario represents a moderate level of potential survival benefits. Scenarios 4 and 5: For the placebo group, we selected the lognormal distribution (representing the best potential survival benefit) and Gompertz distribution (representing the worst potential survival benefit). Scenarios 6 and 7: As penpulimab has not yet been included in the national reimbursement drug list, we evaluated its potential cost-effectiveness under different reimbursement scenarios. Specifically, we analyzed the impact of price reductions of 85% and 60% under a hypothetical future reimbursement policy. These scenarios help to assess the potential accessibility of penpulimab under different pricing strategies.

Health utility values, often critical in determining the incremental cost-effectiveness ratio (ICER), were evaluated in additional scenario analyses using alternative sources. Scenario 8 used utility values from Nafees et al. (2017), specific to Chinese patients, assigning 0.815 for the PFS state and 0.321 for the PD state. Scenario 9 applied utility values from Chouaid et al. (2013), representing patients in Europe and Canada, with 0.71 for the PFS state and 0.67 for the PD state.

To evaluate the effects of different time horizons on the analysis, the base case was conducted using a 10-year time frame. In addition, three alternative scenarios were considered to examine the impact of shorter and longer durations: scenario 10 explored a 5-year horizon, scenario 11 extended the analysis to 15 years, and scenario 12 further expanded it to a 20-year period.

## 3 Result

### 3.1 Base-case analysis

The results of the base-case analysis are summarized in Table 4. Regarding health outcomes, patients in the penpulimab group gained 0.86 more life-years (LYs) than the placebo group, with an increase of 0.75 QALYs after accounting for quality of life. Regarding costs, the total treatment cost for the penpulimab group was $38,297.01, while the price for the placebo group was $26,203.04. Therefore, the ICER for the penpulimab group compared to the placebo group was $16,105.90/QALY. At a willingness-to-pay (WTP) threshold of 3 times China’s 2023 *per capita* gross domestic product (GDP), which is $37,709.46 per QALY (National Bureau of Statistics, 2024b), penpulimab plus paclitaxel and carboplatin is more cost-effective than placebo combined with paclitaxel and carboplatin for first-line treatment of locally advanced or metastatic NSCLC.

**TABLE 4:** Base-case results.

Category	Penpulimab group	Placebo group	Incremental
Costs ($)	38,297.01	26,203.04	12,093.97
QALYs	2.73	1.98	0.75
LYs	3.37	2.50	0.86
ICER ($/QALY)			16,105.90

ICER: incremental cost-effectiveness ratio; LYs: life-years; QALYs, quality-adjusted life-years.

### 3.2 One-way deterministic sensitivity analysis

**FIGURE 5:**
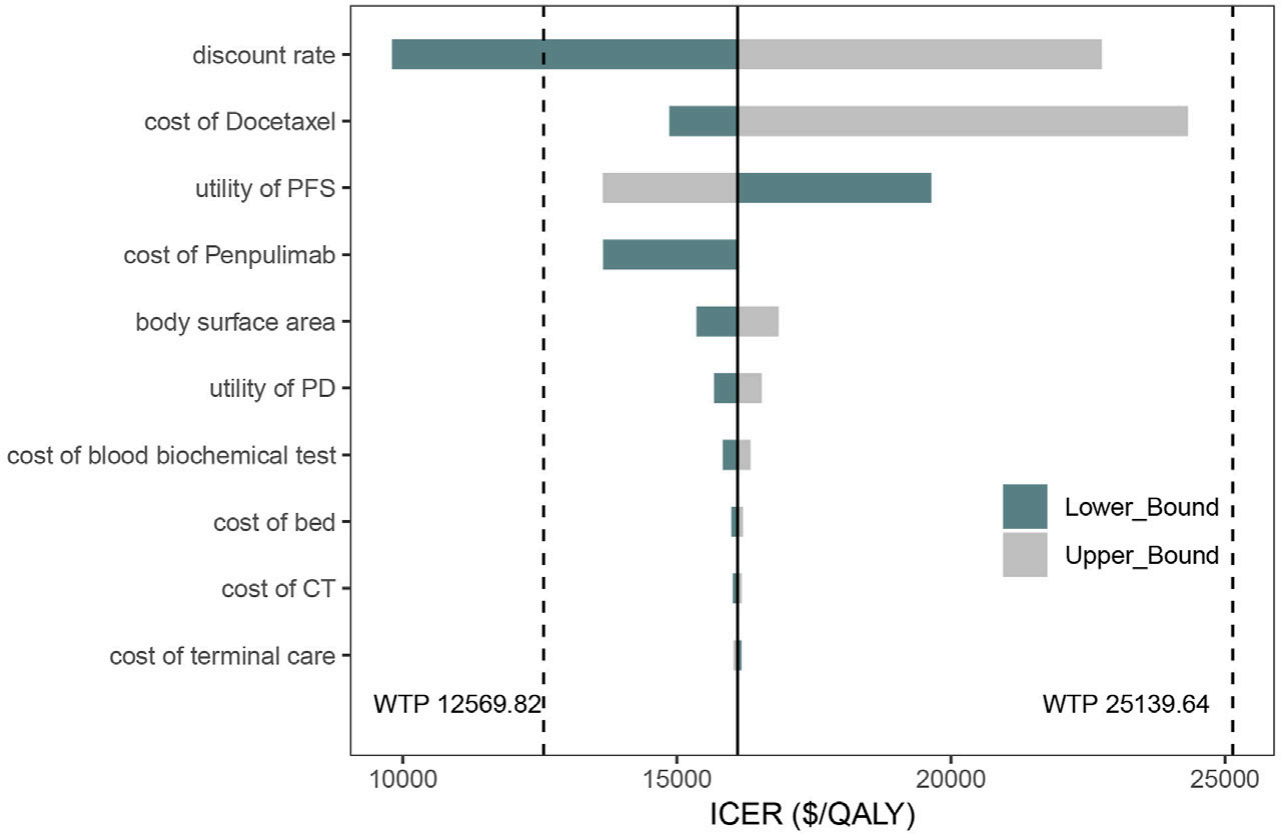
Tornado diagram of one-way deterministic sensitivity analysis.

The results are presented in Figure 5. The tornado diagram displays the top 10 parameters most significantly impacting the results. Key factors influencing the outcomes include the discount rate, the cost after disease progression, the utility values for PFS and PD states, penpulimab, and the body surface area. Despite variations in all uncertain parameters, the ICER remains under 3 times the *per capita* GDP, demonstrating the robustness of the base-case analysis results.

### 3.3 Probabilistic sensitivity analysis

**FIGURE 6:**
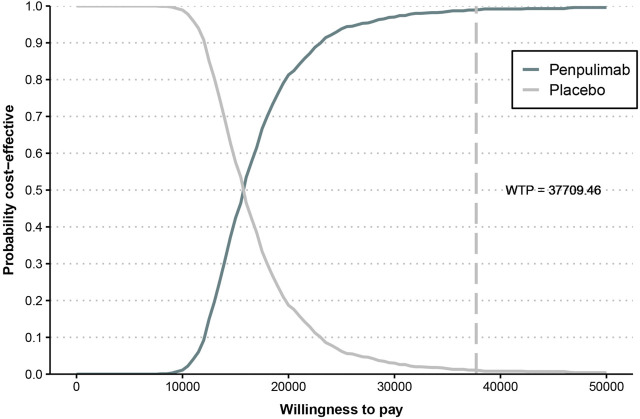
Cost-effectiveness acceptability curve.

**FIGURE 7:**
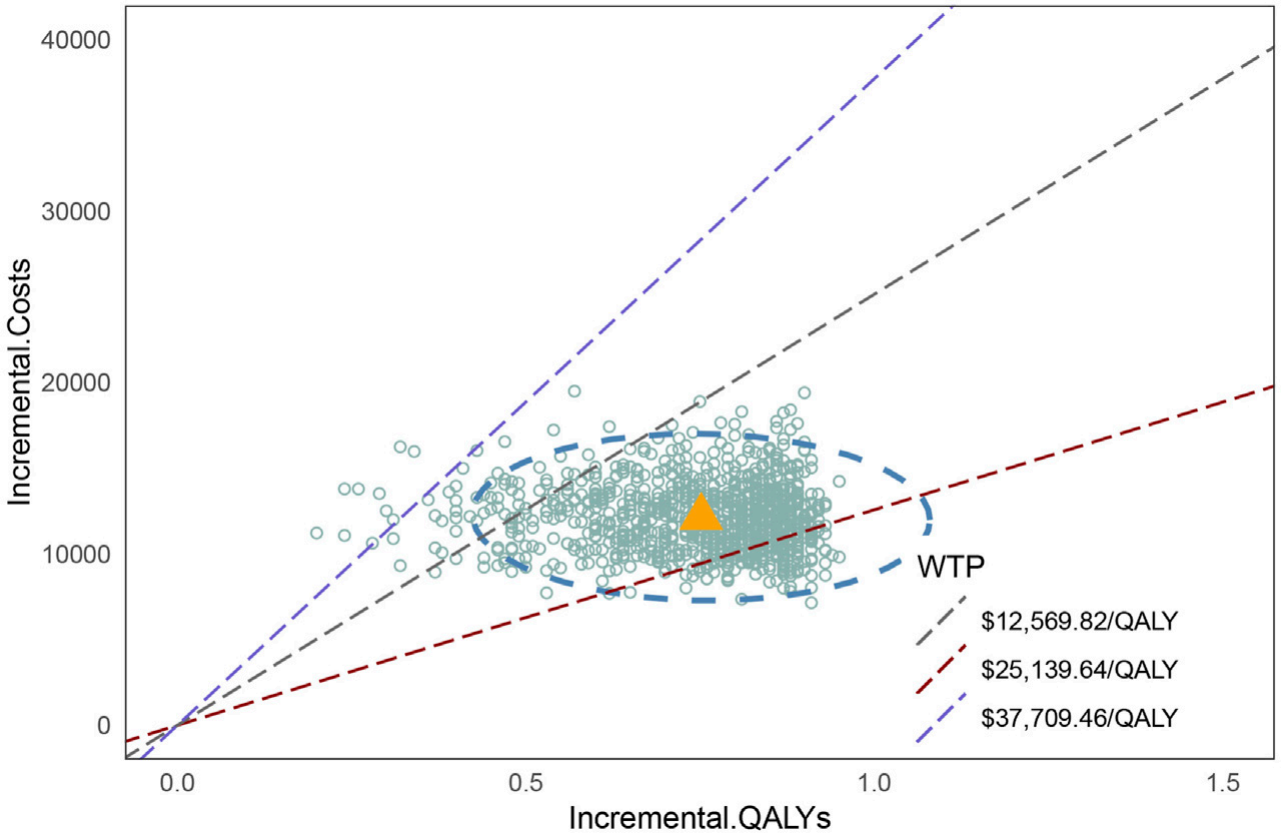
Scatter plot of the cost-effectiveness plane..

The cost-effectiveness acceptability curve (Figure 6) demonstrates that the cost-effectiveness of the penpulimab group increases with a higher WTP threshold. When the WTP threshold reaches approximately $16,000, the probabilities of both cost-effective treatments are similar, at 50%. When the WTP threshold was $37,709.46, the probability of penpulimab being cost-effective reached 99%. The scatter plot of the incremental cost-effectiveness plane is shown in Figure 7. At the WTP thresholds of 1 time and 2 times the 2023 Chinese *per capita* GDP ($12,569.82/QALY and $25,139.64/QALY, respectively), 15.3% and 94.1% of the simulations are cost-effective. The results of the probabilistic sensitivity analysis align with the base-case analysis, confirming the robustness of the model.

### 3.4 Scenario analyses

The scenario analyses are presented in Table 5. The results indicate that when the Gompertz distribution was used for extrapolation in the penpulimab group, ICER reached $62,250 per QALY, significantly exceeding the predefined WTP threshold, thereby rendering penpulimab no longer cost-effective. However, none of the other parametric models altered overall cost-effectiveness.

**TABLE 5:** Cost-effectiveness in scenario analyses.

	Plan	Incremental cost	Incremental QALY	ICER
Scenario 1	Penpulimab OS curve (lognormal distribution)	13,015.61	0.95	13,755.10
Scenario 2	Penpulimab OS curve (Gompertz distribution)	9246.98	0.15	62,250.41
Scenario 3	Penpulimab OS curve (Weibull distribution)	10,329.54	0.37	27,948.89
Scenario 4	Placebo OS curve (lognormal distribution)	10,647.97	0.65	16,480.70
Scenario 5	Placebo OS curve (Gompertz distribution)	18,643.59	1.23	15,120.63
Scenario 6	Penpulimab price reduced to 85%	10,712.50	0.75	14,266.16
Scenario 7	Penpulimab price reduced to 60%	8410.06	0.75	11,199.93
Scenario 8	PFS: 0.815, PD: 0.321	12,093.97	0.76	15,860.85
Scenario 9	PFS: 0.71, PD: 0.67	12,093.97	0.62	19,531.48
Scenario 10	5-year time horizon	12,965.79	0.48	27,005.02
Scenario 11	15-year time horizon	12,041.36	0.86	14,000.63
Scenario 12	20-year time horizon	12,083.64	0.91	13,336.70

ICER:incremental cost-effectiveness ratio; QALYs: quality-adjusted life-years; PFS: progression-free survival; PD: progression disease.

In Scenarios 6 and 7, we explored price reductions to 85% and 60%, respectively, under insurance reimbursement. These findings suggest that as the price of penpulimab decreases, the ICER also declines, increasing its likelihood of being cost-effective. Scenarios 8 and 9 examined the impact of lower PFS and PD utility values, leading to an increase in ICER. However, the results remained within one to two times the *per capita* GDP, without reversing the cost-effectiveness conclusion. In Scenario 10, when the time horizon was shortened to 5 years, both the incremental cost and QALYs decreased, but the ICER increased to $27,005.02 per QALY. Conversely, when the time horizon was extended to 15 and 20 years, both incremental cost and QALYs increased, while the ICER declined, emphasizing the long-term benefits of penpulimab.

Overall, the scenario analyses further validated the robustness of the base-case results, reinforcing the cost-effectiveness of penpulimab under most modeled conditions.

## 4 Discussion

Penpulimab is the first humanized immunoglobulin G1 (IgG1) monoclonal antibody independently developed in China, with high affinity and binding specificity for PD-1 (Huang et al., 2022). It was approved in 2023 for the first-line treatment of locally advanced or metastatic squamous NSCLC. However, penpulimab is not currently included in the Chinese National Reimbursement Drug List, meaning that despite its proven efficacy in clinical trials, patients must pay out-of-pocket, as the national medical insurance system does not cover it. Several studies have evaluated the economic aspects of PD-1/PD-L1 inhibitors. Shao et al. (2022) compared the cost-effectiveness of camrelizumab plus chemotherapy versus chemotherapy alone in patients with advanced squamous NSCLC, reporting an ICER of approximately $13,572 per QALY, suggesting an economic advantage of camrelizumab in combination with chemotherapy. Similarly, Liu et al. (2022) assessed the cost-effectiveness of sintilimab combined with pemetrexed and platinum-based chemotherapy compared to chemotherapy alone, finding an ICER of approximately CNY ¥220,963.22 per QALY.

In contrast, this study is the first to evaluate the cost-effectiveness of penpulimab, providing economic evidence based on clinical data from the AK105-302 trial. The base-case analysis results show that the incremental cost and incremental QALY are $12,093.97 and 0.75 QALY, respectively, with an ICER of $16,105.90/QALY, which is well below the WTP threshold of 3 times China’s *per capita* GDP. The study found that the ICER of penpulimab, compared to chemotherapy alone, is similar to that of other PD-1/PD-L1 inhibitors. Although penpulimab has not yet been included in national reimbursement drug list, it has already demonstrated potential economic value. Additionally, this study conducted an exploratory analysis of price reductions under insurance coverage. The results showed that when the cost of penpulimab was reduced to 85% and 60%, its ICER values were $14,266.16/QALY and $11,199.93/QALY, respectively. These findings may serve as a reference for future negotiations on national reimbursement inclusion.

The parameters in this study represent all key inputs in the partitioned survival model, including drug acquisition costs for penpulimab, paclitaxel, carboplatin, and docetaxel, as well as utility values, transition probabilities, and adverse event costs. However, the tornado diagram reveals that the costs of penpulimab, paclitaxel, and carboplatin, central to the AK105-302 trial, have a minimal impact on model outcomes compared to the cost of docetaxel. This discrepancy can be attributed to the treatment design and clinical dynamics modeled in the analysis. Penpulimab, paclitaxel, and carboplatin are administered in the PFS state, which has a relatively shorter duration in the trial. In contrast, docetaxel is used as a second-line treatment in the PD state, which typically lasts longer. Consequently, the cumulative costs associated with docetaxel treatment over an extended PD period disproportionately influence ICER.

Moreover, the high variability in docetaxel pricing, ranging from $3.18 to $128.01 per 20 mg dose, significantly impacts model outcomes. This variability reflects the substantial cost difference between branded and generic formulations in the Chinese market. On the other hand, the costs of penpulimab, paclitaxel, and carboplatin were derived from uniform, median bid prices, which exhibit less variation. As a result, the sensitivity of ICER to fluctuations in these costs is reduced.

Although the influence of docetaxel costs raises questions about the stability of the model conclusions, it is important to note that the ICER for penpulimab consistently remained below the WTP threshold of three times the *per capita* GDP across sensitivity analyses. This highlights the conclusion’s robustness, even under varying cost assumptions. Further research is warranted to explore the potential impact of alternative second-line treatments and reimbursement policies on model outcomes.

In the one-way sensitivity analysis, the primary driver of the ICER is the treatment cost after disease progression. In the AK105-302 clinical trial, it was reported that patients in the placebo group could transition to open-label penpulimab monotherapy after confirmed disease progression, with 102 placebo group patients crossing over to receive penpulimab (Zhong et al., 2024). Additionally, patients in the penpulimab group could continue receiving penpulimab after disease progression if deemed clinically beneficial. Due to the limitations in the original data, we assumed that treatment crossover in the placebo group would not impact patient survival. The cost calculation includes the crossover to penpulimab treatment and guideline-recommended second-line docetaxel treatment (Chinese Society of Clinical Oncology, 2024; Oncology Society of Chinese Medical Association, 2024). However, in actual clinical practice, the OS rate of patients in the placebo group may be influenced by the treatment switch, potentially introducing some bias into the analysis (Latimer and Abrams, 2014).

For the penpulimab group, the proportion of patients continuing penpulimab treatment after disease progression was not explicitly mentioned. Therefore, we conservatively assumed that all patients would continue receiving penpulimab after disease progression. As the cost of second-line docetaxel used in the base-case analysis is significantly lower than the cost of penpulimab, this assumption may result in overestimating the total cost for the penpulimab group, potentially affecting the ICER. However, this is unlikely to lead to a reversal of the results.

In the one-way sensitivity analysis, the utility values for PFS and PD impact the ICER. In the base-case analysis, we used utility values from Shen et al. (2018) based on data from Chinese NSCLC patients, which showed that the incremental QALY for the penpulimab group was 0.75 compared to the placebo group. In the scenario analysis, we applied utility values from Nafees et al. (2017) for Chinese patients and Chouaid et al. (2013) for Western patients. The incremental QALYs were 0.76 and 0.62, respectively, and the ICER results did not exceed the WTP threshold.

Owing to the immature OS data for the penpulimab group in the AK105-302 trial, with no median OS reached, we selected parameter distributions representing both the best and worst potential survival benefits. The results indicated that while the ICER for the placebo group remained stable across all models, the ICER for the penpulimab group varied from $13,755.10 per QALY to $62,250.41 per QALY. This variation suggests that the economic value of penpulimab is highly dependent on its long-term survival benefit, which remains uncertain owing to the lack of median OS data.

Nevertheless, we further fitted and extrapolated survival data using other alternative distribution models, and the results showed that ICER values remained within the WTP threshold range, indicating a certain degree of robustness in our findings. However, given the absence of publicly available long-term survival data for external validation, the results of this study should be cautiously interpreted. Future long-term follow-up data on penpulimab are essential to further validate our findings and optimize the reliability of the economic model.

The one-way sensitivity analysis found that the primary driver is the docetaxel cost. Since the AK105-302 trial did not specify the second-line treatment for patients after disease progression, we assumed that patients would receive docetaxel-based second-line therapy according to guideline recommendations. This assumption may introduce some bias. The significant impact of docetaxel on the study results can be attributed to the long duration of the PD state and the considerable cost difference between original and generic drugs in China. This results in substantial fluctuations in the cost of docetaxel in the one-way sensitivity analysis. In this study, the cost of a single 20 mg dose of docetaxel ranges from $3.18 to $128.01, causing the per-cycle cost to vary between $20.51 and $825.66. The analysis reveals that as the cost of docetaxel increases, the cost-effectiveness of the penpulimab group declines. According to the guideline for the treatment of NSCLC, recommended second-line treatments for stage IV non-driver gene squamous NSCLC include nivolumab, tislelizumab, or docetaxel monotherapy (Chinese Society of Clinical Oncology, 2024). However, current economic studies on second-line therapies for advanced NSCLC in the Chinese population indicate that nivolumab has not shown economic advantages (Zhou et al., 2022). In the one-way sensitivity analysis, the highest docetaxel cost exceeded the per-cycle cost of tislelizumab. However, the cost-effectiveness of penpulimab as a first-line treatment remains robust and below the 3 times the *per capita* GDP threshold. In this study, the ICER positively correlates with the cost of penpulimab, meaning the cost reduction would improve cost-effectiveness.

Due to regional disparities in China, *per capita* GDP varies significantly across provinces. Using 3 times the 2023 regional *per capita* GDP as the WTP threshold, economically developed regions like Beijing and Shanghai have a WTP exceeding $80,000/QALY, while areas such as Gansu and Heilongjiang have a WTP below $30,000/QALY (National Bureau of Statistics, 2024a). These differences may lead to a reversal in the cost-effectiveness results for penpulimab. Therefore, regional economic differences should be considered in clinical practice to enable reasonable resource allocation.

The AK105-302 trial also conducted subgroup analyses. The results showed no statistically significant difference in PFS between the two groups in patients with clinical stages (IIIb or IIIc) and non-smokers. Additionally, there was no statistically significant difference in OS between the two groups in patients aged under 65, with an ECOG score of 0, clinical stages (IIIb or IIIc), never smokers, and those with a PD-L1 tumor proportion score of less than 1% (Zhong et al., 2024). In these subgroups, the survival benefit of the penpulimab group compared with that of the placebo group was limited. Consequently, the incremental QALYs decreased, leading to a higher ICER that, in some cases, could exceed the WTP threshold, particularly among non-smokers and patients with clinical stage IIIb or IIIc disease. These findings suggest that penpulimab may not provide a cost-effectiveness advantage over placebo in certain sub-populations. However, owing to the absence of PFS and OS data specific to these subgroups in the trial, detailed subgroup analyses were not conducted. Further research is needed to confirm the cost-effectiveness of penpulimab in combination with paclitaxel and carboplatin in these patients.

This study also has several limitations. First, the current model assumes penpulimab is used for no more than 2 years (Zhong et al., 2024). If penpulimab is used for more than 2 years, treatment costs will increase, but the survival benefits may not increase proportionally, which could slightly increase the ICER value (Dai et al., 2023). Second, the data used in this study were reconstructed pseudo-individual data from software, which may introduce some bias in the results. Third, since the AK105-302 trial did not include a patient preference study for utility values, we used utility values based on Chinese patients and conducted scenario analyses with different utility values. However, some deviations from real-world situations may still exist. Fourth, this study did not consider adverse events in grades 1 and 2. Although sensitivity analysis showed minimal adverse event costs and incidence impact on the results, it may still introduce some bias.

## 5 Conclusion

This study evaluates the cost-effectiveness of penpulimab combined with paclitaxel and carboplatin compared to placebo combined with paclitaxel and carboplatin as a first-line treatment for metastatic or advanced squamous NSCLC patients in China. From the perspective of the Chinese healthcare system, the base-case analysis shows that the ICER for penpulimab is $16,105.90/QALY, which is below 3 times the 2023 *per capita* GDP of China ($37,709.46). These results suggest that penpulimab may be a cost-effective option compared with placebo. However, given the immature overall survival data and the outcomes of the sensitivity analyses, these findings should be interpreted with caution. Further validation using long-term follow-up data is necessary to confirm the economic value of penpulimab. Nonetheless, this study provides valuable insights into local clinical decision making.

## Data Availability

The raw data supporting the conclusions of this article will be made available by the authors, without undue reservation.

## References

[B1] Blandin K. S. Crosbie P. A. Balata H. Chudziak J. Hussell T. Dive C. (2017). Progress and prospects of early detection in lung cancer. Open Biol. 7, 170070. 10.1098/rsob.170070 28878044 PMC5627048

[B2] Cheng M. Shao Y. Li L. Jiang M. Song Z. (2024). Cost-effectiveness of immunotherapies for advanced squamous non-small cell lung cancer: a systematic review. BMC Cancer 24, 312. 10.1186/s12885-024-12043-w 38448878 PMC10916025

[B3] Chinese Pharmaceutical Association (2024). China guidelines for pharmacoeconomic evaluation. Available online at: https://www.cpa.org.cn/cpadmn/attached/file/20201203/1606977380634185.pdf (Accessed November 7, 2024).

[B4] Chinese Society of Clinical Oncology (2023). Chinese society of clinical Oncology (CSCO) Diagnosis and treatment guidelines for non-small cell lung cancer (NSCLC), 2023. Beijing: People’s Medical Publishing House.

[B5] Chinese Society of Clinical Oncology (2024). Chinese society of clinical Oncology (CSCO) Diagnosis and treatment guidelines for non-small cell lung cancer (NSCLC), 2024. Beijing: People’s Medical Publishing House.

[B6] Chouaid C. Agulnik J. Goker E. Herder G. J. Lester J. F. Vansteenkiste J. (2013). Health-related quality of life and utility in patients with advanced non-small-cell lung cancer: a prospective cross-sectional patient survey in a real-world setting. J. Thorac. Oncol. 8, 997–1003. 10.1097/JTO.0b013e318299243b 23787802

[B7] Dai H. Wang W. Fan X. Chen Y. (2023). Cost-effectiveness of camrelizumab plus chemotherapy vs. chemotherapy in the first-line treatment of non-squamous NSCLC: evidence from China. Front. Med. (Lausanne) 10, 1122731. 10.3389/fmed.2023.1122731 36865055 PMC9971596

[B8] Han B. Zheng R. Zeng H. Wang S. Sun K. Chen R. (2024). Cancer incidence and mortality in China. J. Natl. Cancer Cent. 4, 47–53. 10.1016/j.jncc.2024.01.006 39036382 PMC11256708

[B9] Herbst R. S. Morgensztern D. Boshoff C. (2018). The biology and management of non-small cell lung cancer. Nature 553, 446–454. 10.1038/nature25183 29364287

[B10] Huang Z. Pang X. Zhong T. Qu T. Chen N. Ma S. (2022). Penpulimab, an fc-engineered IgG1 anti-PD-1 antibody, with improved efficacy and low incidence of immune-related adverse events. Front. Immunol. 13, 924542. 10.3389/fimmu.2022.924542 35833116 PMC9272907

[B11] Latimer N. R. (2013). Survival analysis for economic evaluations alongside clinical trials--extrapolation with patient-level data: inconsistencies, limitations, and a practical guide. Med. Decis. Mak. 33, 743–754. 10.1177/0272989X12472398 23341049

[B12] Latimer N. R. Abrams K. R. (2014). NICE DSU technical support document 16: adjusting survival time estimates in the presence of treatment switching. London: National Institute for Health and Care Excellence NICE. 27466662

[B13] Liu H. Wang Y. He Q. (2022). Cost-effectiveness analysis of sintilimab plus pemetrexed and platinum versus chemotherapy alone as first-line treatment in metastatic non-squamous non-small cell lung cancer in China. Health Econ. Rev. 12, 66. 10.1186/s13561-022-00410-x 36581793 PMC9801637

[B14] Nafees B. Lloyd A. J. Dewilde S. Rajan N. Lorenzo M. (2017). Health state utilities in non-small cell lung cancer: an international study. Asia Pac J. Clin. Oncol. 13, e195–e203. 10.1111/ajco.12477 26990789

[B15] National Bureau of Statistics (2024a). Local government website. Available online at: https://www.stats.gov.cn/sj/ (Accessed November 7, 2024).

[B16] National Bureau of Statistics (2024b). Statistical communiqué of the people's Republic of China on the 2023 national economic and social development. Available online at: https://www.stats.gov.cn/sj/zxfb/202402/t20240228_1947915.html (Accessed November 7, 2024).

[B17] National Comprehensive Cancer Network (2024). National Comprehensive Cancer Network (NCCN) clinical practice guidelines in oncology: non-small cell lung cancer version 7. 2024. Available online at: https://www.nccn.org/ (Accessed November 7, 2024). 10.6004/jnccn.2204.0023 38754467

[B18] Oncology Society of Chinese Medical Association (2024). Chinese medical association guideline for clinical Diagnosis and treatment of lung cancer (2024 edition). Chin. J. Oncol. 46, 805–843. 10.3760/cma.j.cn112152-20240510-00189 39045625

[B19] Rothwell B. Kiff C. Ling C. Brodtkorb T. H. (2021). Cost effectiveness of nivolumab in patients with advanced, previously treated squamous and non-squamous non-small-cell lung cancer in England. Pharmacoecon Open 5, 251–260. 10.1007/s41669-020-00245-4 33332018 PMC8160043

[B20] Rui M. Fei Z. Wang Y. Zhang X. Ma A. Sun H. (2022). Cost-effectiveness analysis of sintilimab + chemotherapy versus camrelizumab + chemotherapy for the treatment of first-line locally advanced or metastatic nonsquamous NSCLC in China. J. Med. Econ. 25, 618–629. 10.1080/13696998.2022.2071066 35475459

[B21] Shao T. Ren Y. Zhao M. Tang W. (2022). Cost-effectiveness analysis of camrelizumab plus chemotherapy as first-line treatment for advanced squamous NSCLC in China. Front. Public Health 10, 912921. 10.3389/fpubh.2022.912921 36045725 PMC9423383

[B22] Shen Y. Wu B. Wang X. Zhu J. (2018). Health state utilities in patients with advanced non-small-cell lung cancer in China. J. Comp. Eff. Res. 7, 443–452. 10.2217/cer-2017-0069 29775084

[B23] State Administration of Foreign Exchange (2024). RMB central exchange rate. Available online at: https://www.safe.gov.cn/safe/rmbhlzjj/index.html (Accessed November 7, 2024).

[B24] Su D. Wu B. Shi L. (2021). Cost-effectiveness of atezolizumab plus bevacizumab vs sorafenib as first-line treatment of unresectable hepatocellular carcinoma. JAMA Netw. Open 4, e210037. 10.1001/jamanetworkopen.2021.0037 33625508 PMC7905498

[B25] Surveillance Research Program, National Cancer Institute (2024). SEER*Explorer: an interactive website for SEER cancer statistics. Available online at: https://seer.cancer.gov/statistics-network/explorer/ (Accessed November 7, 2024).

[B26] Woodard G. A. Jones K. D. Jablons D. M. (2016). Lung cancer staging and prognosis. Cancer Treat. Res. 170, 47–75. 10.1007/978-3-319-40389-2_3 27535389

[B27] Woods B. S. Sideris E. Palmer S. Latimer N. Soares M. (2020). Partitioned survival and state transition models for healthcare decision making in oncology: where are we now? Value Health 23, 1613–1621. 10.1016/j.jval.2020.08.2094 33248517

[B28] Xiang H. Meng K. Wu M. Tan C. (2024). Cost-effectiveness analysis of first-line serplulimab plus chemotherapy for advanced squamous non-small-cell lung cancer in China: based on the ASTRUM-004 trial. Expert Rev. Pharmacoecon Outcomes Res. 24, 1043–1051. 10.1080/14737167.2024.2379600 38984534

[B29] Zhong H. Sun S. Chen J. Wang Z. Zhao Y. Zhang G. (2024). First-line penpulimab combined with paclitaxel and carboplatin for metastatic squamous non-small-cell lung cancer in China (AK105-302): a multicentre, randomised, double-blind, placebo-controlled phase 3 clinical trial. Lancet Respir. Med. 12, 355–365. 10.1016/S2213-2600(23)00431-9 38309287

[B30] Zhou D. Luo X. Zhou Z. Zeng X. Wan X. Tan C. (2022). Cost-effectiveness analysis of tislelizumab, nivolumab and docetaxel as second- and third-line for advanced or metastatic non-small cell lung cancer in China. Front. Pharmacol. 13, 880280. 10.3389/fphar.2022.880280 36091746 PMC9453816

